# Comparison of Scrotal Orchiopexy Versus Traditional Inguinal Orchiopexy for Palpable Undescended and Retractile Testis in Children: Insights from a Greek Surgical Center

**DOI:** 10.3390/life16020360

**Published:** 2026-02-21

**Authors:** Maria Florou, Triantafyllia Koletsa, Sophia Tsokkou, Georgia Raptou, Antonia Syrnioti, Ioannis Spyridakis, Christos Kaselas

**Affiliations:** 1 Second Department of Pediatric Surgery, Aristotle University of Thessaloniki, “Papageorgiou” General Hospital, 564 03 Thessaloniki, Greece; ispyrida@auth.gr (I.S.);; 2 Department of Pathology, School of Medicine, Aristotle University of Thessaloniki, 546 36 Thessaloniki, Greece; tkoletsa@auth.gr (T.K.); graptou@auth.gr (G.R.); tonia.syrt@gmail.com (A.S.)

**Keywords:** children, cryptorchidism, guidelines, diagnosis, orchiopexy

## Abstract

**Introduction/Purpose**: Congenital cryptorchidism and retractile testis represent the most commonly presented abnormalities of the male genitourinary system. Orchiopexy is the surgical treatment for both conditions and can be performed either via the conventional two-incision surgical approach or via a singular scrotal incision. The present study firstly investigated the complications associated with each orchiopexy approach in a single-center pediatric surgical department and secondly compared the surgical outcomes in children with congenital cryptorchidism or retractile testes. **Methodology**: A retrospective analysis was conducted in pediatric patients with either congenital cryptorchidism or retractile testes who underwent orchiopexy from 2015 to 2019. Data collected during the study included diagnosis, surgical technique and both short- and long-term complications. Patient stratification was performed in accordance with the type of orchiopexy and the diagnosis UDT vs. RT and inguinal orchidopexy vs. scrotal orchidopexy. **Results**: A total of 362 children underwent 443 orchiopexies of which 227 were inguinal and 216 were scrotal. Complications were reported in 16 (3.6%) surgeries and from which 14 (3.16%) were presented in for postoperative complications. Short-term complications were presented in four (0.9%) cases and consisted of wound dehiscence. Long-term complications were recorded in 12 (2.7%) cases, including recurrence in six (1.3%) testes, testicular atrophy in three (0.6%) cases and presentation of hydrocele or inguinal hernia in two (0.4%) and one (0.2%) patient, respectively. Notably, 13 orchidopexies with preoperative diagnosis of congenital cryptorchidism, were linked with complications (*p* = 0.01), 12 of which underwent with two-incision technique (*p* = 0.07). **Conclusions**: The findings of the study suggest that a preoperative diagnosis of congenital cryptorchidism is a possible risk factor for postoperative complications. With regard to the surgical technique performed, the single-incision scrotal orchiopexy appears to be a much safer and more effective approach palpable undescended testes, compared to the two-incision approach. For the high-lying testes, although the single-scrotal technique provides good results, more prospective studies with selected impalpable undescended testes are needed to strengthen the existing literature.

## 1. Introduction

### 1.1. What Are Undescended Testes and Retractile Testes?

Undescended testes (UDT), also known as cryptorchidism, refers to the testis that has not completed a physiological descent into the scrotum, remaining instead in the inguinal canal or higher, whereas retractile testes (RT) are those that can be manipulated into the scrotum but may reside outside due to the cremasteric reflex [1,2,3].

It is an important distinction that the RT is differentiated from the UDT, since it has completed its normal descent into the scrotum previously at birth. At times, an RT might spontaneously be found in the suprascrotal position along the normal line of descent and can be manipulated back into the scrotum, where it remains until the cremaster reflex is stimulated. The cremaster reflex is highly pronounced between the second and 12th year of life, with a prevalence for RT among the school-aged boys being approximately 3.9% [4,5]. The clinical significance arises from the fact that one-third of the RT cases potentially develop into acquired UDT and thus require monitoring on a regular basis until adolescence, in order to identify the testicular ascent and the need for orchiopexy.

Because of this risk, annual physical examination is recommended to monitor for any upward migration of the testis. Any clinical change observed during re-examination is considered an indication for surgery. Specifically, three signs during physical examination are considered indications for surgery, known as Wyllie’s criteria, and are as follows: (i) the size of the examined testicle is smaller than the contralateral one, (ii) tension in the spermatic cord causing pain to the child when the testicle is pulled into the hemiscrotum, and (iii) immediate retraction of the testicle out of the hemiscrotum. The treatment of retractile testis is the same as for UDT, including orchiopexy, either inguinal or scrotal [6,7].

### 1.2. Epidemiology and Prevalence of Cryptorchidism

Epidemiologically, the congenital undescended testis in children occurs 1 to 4% of males born at term or early term and 45% in male infants born pre-term. Out of the total cases of cryptorchidism, around 80% appear to be palpable upon physical examination. In the majority of cases, the UDT is unilateral, with a higher prevalence seen in the right testes due to embryological development [1,8,9].

Furthermore, retractile testes are less precisely qualified in population studies, but among males referred for testicular malposition, RT accounts for a substantial proportion, with some single-centered studies reporting up to 39% of cases. RT is commonly exhibited in prepubertal boys, and it is considered a variant of palpable testes. Usually, RTs resolve spontaneously by the time of puberty, with rates reported as 75% of cases, but 30 to 32% may become acquired UDT, particularly boys in the age group of 7 years and below [1,10,11,12].

### 1.3. Diagnosis and Treatment of Cryptorchidism

The American Urology Association (AUA) and the Canadian Urological Association-Pediatric Urologists of Canada (CUA-PUC) recommend that the diagnosis be established between the third and sixth months of life, as a spontaneous descent after the sixth month is unlikely. When it comes to infants with UDT persisting beyond the sixth month, a surgical intervention is mandated to prevent lifelong complications [13,14]. The rationale for this timing is that continued non-scrotal position after 6 months is linked with a low probability of spontaneous descent and increased risk of long-term complications. Early surgical correction (orchiopexy) is therefore indicated to optimize long-term outcomes [13,14].

The treatment of choice for the UDT in male infants following spontaneous descent that has not occurred within 6 months is orchiopexy—a surgical repositioning of the testis into the scrotum.

The treatment of choice is orchiopexy, either inguinal, scrotal, or laparoscopic, and preferably it should be scheduled between the 6th and 12th month of the child’s life, and no later than the 18th month of the child’s life. The early surgical correction aims to prevent the long-term complications of cryptorchidism, which are as follows: (i) the reduced fertility in adult life, (ii) the risk of testicular malignancy, (iii) the risk of testicular torsion, and (iv) the psychological effects due to appearance on the person [15].

In our department, orchiopexy for retractile testes is performed only when Wyllie’s criteria are met, ensuring that surgery is reserved for cases at genuine risk of secondary ascent or functional compromise. Specifically, intervention was indicated when serial examinations demonstrated progressive ascent, when persistent cord tension prevented the testis from maintaining a stable scrotal position, when variation in testicular size suggested impaired development, or when the testis could be positioned in the scrotum but immediately retracted, fulfilling the characteristic features described by Wyllie. These criteria guided our selection of RT cases for surgical management and ensured that only clinically significant retractile testes were included in the study [6,7].

The goal of early correction is to minimize the significant risks associated with maintaining the testis in a warmer, non-scrotal environment. When it comes to minimizing the risk of infertility, the testicular exposure to high and non-optimal temperatures (35.1 °C and higher) impairs spermatogenesis, significantly increasing the risk of subfertility or infertility in adulthood, especially in bilateral cases. Orchiopexy effectively reduces the testicular temperature to the scrotal norm, with postoperative measurements showing a decrease to about 34.3 °C, which is associated with improved testicular growth and function [16,17,18,19]. Moving on to the possible development of testicular malignancy, the risk of testicular malignancy in male infants with an undescended testis is approximately 3%, which is five to 10 times greater than that of the general population, and it is linked with an elevated lifetime risk of developing testicular germ cell tumors [1,16,17]. When it comes to testicular torsion, the cryptorchid testicular torsion accounts for 5–15% of cases of testicular torsion.

Although rare in UDT, it is a serious condition requiring emergency management sometimes [20]. Adding on, psychological effects are primarily related to long-term concerns about body image, self-esteem, and anxiety regarding fertility and cancer risk as the child matures. While infants themselves do not experience psychological distress, the diagnosis can cause significant parental anxiety, particularly regarding future reproductive health and malignancy risk [2,17].

### 1.4. Objective of Study

The objective of the present study is to evaluate the clinical significance of primary orchiopexy in pediatric patients diagnosed with congenital UDT or RT and to identify potential risk factors associated with postoperative complications (Table 1). More specifically, the study aims to compare the safety and efficacy of the conventional two-incision inguinal approach with the single-incision scrotal approach and thereby provide evidence-based insights into optimal surgical management for UDT and RT cases.

## 2. Materials and Methods

### 2.1. Study Design and Settings

This study was designed as a retrospective analysis of pediatric patients who underwent orchiopexy, with either the two-incision or single-incision approach at the Second Department of Pediatric Surgery, Papageorgiou General Hospital, Aristotle University of Thessaloniki, between January 2015 and April 2019.

### 2.2. Research Question

The Population, Intervention, Comparator and Outcome (PICO) framework was constructed to outline the research question more clearly.

### 2.3. Patient Selection

All patients who underwent orchiopexy during that timeframe period were screened. The inclusion criteria for each patient were the availability of complete clinical data and a minimum follow-up of 12 months. The exclusion criteria were as follows: (i) orchiopexy performed for indications other than cryptorchism, (ii) prior surgical history in the inguinal region, and (iii) laparoscopic orchiopexy and laparoscopic/inguinal orchiectomy.

### 2.4. Data Collection

Data were extracted from the hospital’s electronic medical records. The following variables were recorded: age at diagnosis, age at surgery, location (bilateral or unilateral), type (true UDT, ectopic, and retractile/ascending), comorbidities, preoperative position of testis, surgical approach (inguinal, scrotal, and laparoscopic), intraoperative position, results, postoperative complications (including atrophy and primary failure) and their management.

### 2.5. Follow-Up and Outcomes Measure

Postoperative follow-up included the following: the immediate assessment of the wound and the healing process, regarding the early complications within 30 days, and for the long-term assessment, the testicular size and position at 6 months and 12 months post-surgery. The complications were divided into two groups: the early complications, which appeared within the first 30 postoperative days, and the late complications, occurring after the first 30 postoperative days.

### 2.6. Statistical Analysis

Data entry and descriptive statistics were performed using the Excel software (Microsoft^®^ Excel version 16.45, 2019 © Microsoft). Continuous variables were tested for normal distribution and expressed as median values. Fisher’s exact test was applied to compare categorical variables and to identify the possible risk factors, such as cryptorchidism and the surgical approach. Statistical analysis was conducted via the SPSS 29 Statistics software, with the *p*-values < 0.05 considered statistically significant.

### 2.7. Ethical Considerations

Written informed consent was obtained from the parents of all pediatric patients prior to surgery.

## 3. Results

A total of 362 pediatric patients underwent orchiopexy between January 2015 and April 2019 in our department, accounting for 471 operated testes. The mean age at operation was 4.35 years (median: age 3 years; range: 6 months to 14 years).

### 3.1. Patients’ Characteristics

Of the 362 patients, 245 (67.7%) were diagnosed with unilateral cryptorchidism and 117 (32.3%) with bilateral cryptorchidism. The majority of the cryptorchid testes (78.5%) were low-lying testes (at mid-low inguinal canal or external ring), and the rest (21.5%) were high-lying (intrabdominal testis, at internal ring or high inguinal canal). Regarding the surgical approach, 227 (48.2%) testes were treated with inguinal orchiopexy, 216 (45.9%) were treated via the scrotal approach, while the 28 cases that received laparoscopic orchiopexy and laparoscopic/inguinal orchiectomy were excluded from the sample. For the diagnostic distribution of the 443 orchidopexies, 224 (57.6%) were classified as congenital UDT and 219 (42.4%) as RT (Table 2).

### 3.2. Postoperative Complications

Complications were documented in 16 orchiopexies (3.6%) involving 14 (3.16%) patients, of whom two had a bilateral complication, eight had a right-sided complication, and six of them had a left-sided complication. Early postoperative complications (≤30 days) were seen in four (0.9%) cases, all due to wound dehiscence. For late complications (>30 days), twelve (2.7%) cases were seen, including six (1.3%) recurrences, three (0.6%) cases of testicular atrophies, two (0.4%) of hydroceles, and one (0.2%) of inguinal hernia (Table 3).

### 3.3. Risk Factor Analysis

**Figure 1:**
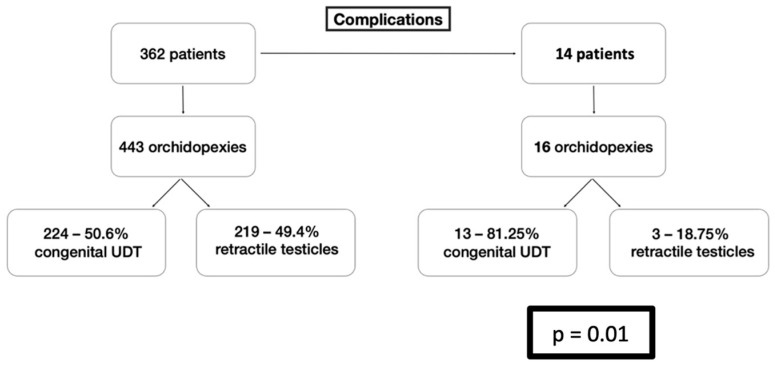
Comparison between the types of Cryptorchidism Diagnosis and Orchidopexy Complications.

Of the 16 testes with reported complications, 13 were diagnosed with congenital UTD and three with RT. The statistical analysis confirmed that congenital UTD was a significant risk factor for complications with a *p*-value of 0.01 (Table 3 and Table 4, and Figure 1).

**Figure 2:**
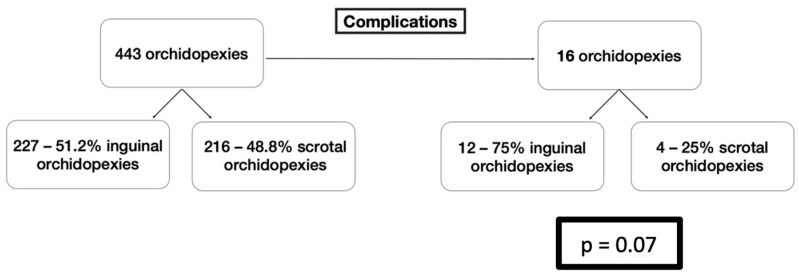
Comparison between the types of Applied Orchidopexy and Complications.

Among the 16 complicated cases, 10 were treated with the inguinal approach and six with the scrotal approach. The differences between the two approaches did not reach a statistically significant difference, with a *p*-value being 0.07 (Figure 2).

## 4. Discussion

The current study investigated the complications of the orchiopexies applied to children with UDT and retractile testicles in one of the busiest Pediatric Surgery departments of Northern Greece. In the latter group, clinical presentation typically occurs after the third year of life, and surgical intervention is preceded by a period of structured observation, in accordance with accepted clinical practice. With regard to UDT, delayed presentation beyond the optimal window for orchidopexy is not uncommon in real-world settings and is largely attributable to late referral patterns from primary care pediatricians and general practitioners. These factors, along with the retrospective design of the study that may reflect older management patterns, are considered responsible for the significantly higher mean surgical age of children at the time of orchidopexy: age 3 years (range: 6 months to 14 years), when compared to international guidelines for UDT alone [8,9,12].

The estimated incidence of complications was 3.9%, in accordance with the literature and within the lower limits. The early complications, occurring within the first 30 days after surgery, were wound dehiscence and the late ones, presenting after the 30th postoperative day, included hydrocele, inguinal hernia, atrophy and recurrence. In the literature the incidence of complications after orchiopexy is estimated from 4% to 11.7% [21,22] and they include the following: (i) hematoma in the wound and/or in the corresponding scrotum, (ii) contamination and the dehiscence of the surgical wound, (iii) atrophy of the operated testicle, (iv) recurrence of cryptorchidism and (v) occurrence of inguinal hernia respectively [22,23].

The most notable observation was the significantly higher complication rate among children with congenital UDT compared with those treated for retractile—acquired undescended testes. This association likely reflects underlying biological differences rather than technical factors alone. Congenital UDT is characterized by disrupted testicular descent, often accompanied by abnormalities of the gubernaculum, cremasteric fibers, and processus vaginalis. These structural deviations may predispose the testis to limited mobilization, increased tension after fixation, or impaired vascular integrity, all of which can contribute to recurrence or atrophy. In contrast, retractile testes have previously completed normal descent and typically retain more favorable anatomy, which may explain their more predictable postoperative course.

After dichotomizing the data based on the preoperative diagnosis and applying the Fischer’s Exact Test, a positive relation was found between the diagnosis of congenital UDT and the presentation of complications (Figure 1). The relation was statistically significant. A possible explanation for this finding may lie in the pathogenesis of these two conditions. Although the testicular descent is a complex process including either genetic, hormonal, environmental, or anatomical factors, no matter what the leading cause is, the presentation of congenital cryptorchidism is always accompanied by the abnormal growth of the gubernaculum and its elements (cremaster muscle, genitofemoral nerve) and the affected development of the processus vaginalis [24,25]. On the other hand, the retractile testis is a clinical condition in which the testes are descended into the scrotum after birth; in later life, and spontaneously, they ascend outside the scrotum, by the cremaster muscle reflex activity, which is induced by the genitofemoral nerve [26]. The phenomenon of retractile testis has traditionally belonged to normal variants, and only a few reports have associated it with inguinal–scrotal anatomical abnormalities, such as a patent processus vaginalis [27]. In the authors’ point of view, the distinct pathogenesis of the congenital cryptorchidism and the retractile—ascending testis, which result in defined differences in inguinal–scrotal anatomy and function, offer a possible explanation for the different occurrence of postoperative complications.

When the testes that suffered a complication were compared on the applied surgical approach, no significant difference was found between the inguinal and the scrotal technique. Our results are in line with the literature, as in many previous studies, the rate of success in these two methods and the rate of complications are similar [28,29].

The conventional inguinal orchiopexy is the gold standard technique, since it offers: (i) sufficient mobilization of the spermatic cord, (ii) separation and high ligation of the processus vaginalis or hernia sac and (iii) the fixation of the testicle in the scrotal pouch without tension [13,30]. In order to decrease the potential morbidity of the inguinal incision, the trans-scrotal orchiopexy was first proposed by Bianchi and Squire and rapidly has become widely accepted among pediatric surgeons for the palpable cryptorchid testis [29,30].

When comparing surgical techniques, both the inguinal and scrotal approaches demonstrated similarly low complication rates, consistent with contemporary literature. The inguinal approach remains widely used because it allows direct visualization of the spermatic cord and facilitates high ligation of a patent processus vaginalis when present. However, the scrotal approach has gained popularity due to its shorter operative time, reduced dissection, and superior cosmetic outcome. In our cohort, the scrotal technique performed comparably to the traditional two-incision method, even though nearly one-fifth of testes were high-lying. The absence of conversions from scrotal to inguinal incision underscores the careful preoperative selection and experience of the surgical team.

The anatomical features of the pediatric population and specifically the short distance from the external to the internal inguinal ring that are almost superimposed, the anteroposterior direction of the inguinal canal and the good mobility of the skin that enables retraction of the skin incision, made the trans-scrotal approach feasible and efficient. Since then, many observational studies followed and verified the benefits of the trans-scrotal approach, including the following: shorter operative times, less dissection of the issues, resulting in less postoperative pain, better cosmetic results and thus, greater patient and surgeon satisfaction [13,31].

On the other hand, there were studies in favor of the inguinal approach, presenting fewer complications than the scrotal technique [32]. When the data was systematically reviewed, the single-scrotal incision appeared sufficient for orchiopexy in patients with a palpable and low-lying UDT, as it offers comparable—if not better—results to the standard two-incision approach [29,33]. Although the majority of the undescended testes are low-lying and near the distal inguinal ring, there is still considerable debate regarding the surgical management and the concurrent complications of the high-lying testes, located within the high inguinal canal and near the inner inguinal ring.

The management of high-lying testes continues to generate debate. Some authors advocate for routine inguinal exploration to ensure adequate cord length and address a potentially patent processus vaginalis, while others report excellent outcomes using a scrotal incision alone. Our findings support the latter perspective: even in cases where the testis was located near the internal ring, the scrotal approach was feasible and did not result in increased recurrence or hernia formation. This aligns with emerging evidence suggesting that routine ligation of the processus vaginalis may not be necessary unless it is clearly patent, and that excessive dissection may not confer additional benefit.

Few studies report their results regarding the complication rates of the inguinal and the scrotal approach, taking into account the preoperative testis location. In 1995, Docimo et al. systematically reviewed orchiopexies with the applied distinction on the preoperative testis location. Their results underline that the more proximal the location of the testis is preoperatively, the more probable a poorer outcome is to occur.

They also estimated an overall recurrence rate at 4.1% [34]. On the contrary, there are studies supporting the scrotal approach even for the high-lying UDT, with good results and low complication rates comparable to the inguinal approach [35]. Gordon’s team in 2005 mentioned that the trans-scrotal technique required a groin incision in 4.4% of the orchiopexies for additional cord length and the overall recurrence rate was 2% [36]. The rate of conversion from one- to two-incision surgical technique widely varies in the literature between 0% and 13% [29].

The recurrence and atrophy rates in our study were low and comparable to those reported internationally. Importantly, the majority of adverse outcomes occurred in children with congenital UDT, reinforcing the need for early diagnosis, timely intervention, and careful intraoperative assessment of cord tension. The low incidence of postoperative hydrocele and inguinal hernia further supports the safety of both techniques when performed by experienced pediatric surgeons.

The lack of distinction of the preoperative testicular locations is a common limitation among studies, including our lack of distinction between high-lying and low-lying testis preoperatively. This phenomenon among studies affects the accuracy of the presented estimations and is probably responsible for the given variability. In the latest meta-analysis by Yu et al. in 2022, the conversion rate is 3.6%, regarding a majority of high-lying intracanalicular testes, and aims to facilitate better dissection of the tissues, adequate length of the spermatic cord and/or high ligation of a patent processus vaginalis [33]. In our study, the 21.5% of the testes were high-lying and were managed by either an inguinal or a scrotal approach, with a zero-conversion rate, reflecting the accuracy of the approach-choice by our surgical team.

Another important point affecting the long-term complications of orchiopexies is the dissection and ligation of the processus vaginalis when found and the need for a scrotal orchiopexy to be converted to inguinal, when a processus vaginalis is found to be either patent or as a fibrous remnant. There is debate whether the one incision orchiopexy could potentially result in postoperative inguinal hernias or hydroceles.

An interesting study by Hyuga et al. investigated the possibility of an inguinal hernia presentation in 137 children (227 testes) operated for UDTs only through the single-scrotal technique. The processus vaginalis was not ligated when found partially patent or as a fibrous remnant, unless it was widely patent. Then, the children were followed up at the same department for approximately 44 months and no inguinal hernia was reported [37]. Similar results with no association between scrotal orchiopexy and metachronous inguinal hernia were presented by Ceccanti et al. [38]. After reviewing the literature, the incidence of patent processus vaginalis in UDTs ranges from 20 to 70% [39], and the occurrence of postoperative inguinal hernias or hydroceles is estimated from 0.7% to 0.9%, resulting in no statistically significant correlation. Putting the pieces together, while performing the single scrotal-incision orchiopexy, the ligation of the processus vaginalis is not necessary, unless it is widely open [31,36,38].

Overall, our results contribute to the growing body of evidence supporting the scrotal approach as a safe and effective alternative to the traditional inguinal technique for palpable testes. The findings also highlight the importance of distinguishing congenital UDT from retractile testes, as the former carries a higher intrinsic risk of postoperative complications regardless of surgical method.

### 4.1. Limitations

The present study has some limitations inherited from its retrospective design. Firstly, the study was conducted at a single center, which may limit the generalizability of findings to other populations and surgical settings. Secondly, although laparoscopic cases were excluded from the comparative analysis, their absence prevents a comprehensive assessment of all available surgical approaches currently used.

### 4.2. Future Perspectives

Future research must focus on prospective, multicenter studies with standardized follow-up protocols to validate these findings. Stratification of patients by preoperative testes position—low-lying versus high-lying—and inclusion of laparoscopic orchiopexy cases would provide a more complete understanding of surgical outcomes. Additionally, long-term studies evaluating fertility potential, hormonal function and psychological outcomes after orchiopexy are warranted to assess the broader impact of surgical management.

In the broader context of pediatric testicular pathology, it is worth noting that advances in diagnostic imaging continue to refine clinical decision-making and postoperative assessment. Recent systematic reviews and meta-analyses evaluating contrast-enhanced ultrasound (CEUS) have demonstrated its high diagnostic accuracy, by presenting particularly strong positive predictive value for identifying neoplastic lesions and excellent negative predictive value for excluding malignancy, underscoring its potential as a minimally invasive, real-time, and cost-effective adjunct to conventional ultrasound. Although these findings pertain primarily to adult populations, they highlight the evolving role of advanced ultrasonographic techniques in testicular evaluation. As imaging modalities become more precise, they may further support early diagnosis, postoperative surveillance, and the differentiation of complications following orchiopexy, especially in cases where testicular viability or vascular integrity is uncertain [40].

## 5. Conclusions

In conclusion, our study highlights that those children operated for congenital undescended testes exhibited a higher rate of postoperative complications compared with those undergoing orchiopexy for retractile—ascending testes that met strict clinical criteria for surgical intervention. This observation reflects differences in underlying anatomy and pathophysiology rather than suggesting that congenital cryptorchidism itself is an independent “risk factor” in the broader population of patients undergoing orchiopexy. Regarding surgical technique, the single-incision scrotal approach proved to be a safe and effective option for palpable testes, demonstrating outcomes comparable to the traditional inguinal method. Further prospective, multicenter studies, particularly those stratifying by preoperative testicular position and including laparoscopic cases, are needed to refine the optimal management of high-lying and impalpable testes. Overall, our findings support the use of scrotal orchiopexy as a reliable alternative to the inguinal approach in appropriately selected pediatric patients.

## Figures and Tables

**Table 1:** The PICO Framework outlines the research question of the study.

Element	Definition in Study	Details
P	Children with congenital cryptorchidism or retractile/ascending testis	Pediatric patients (ages 6 months to 16 years) treated at a Greek surgical center between 2015 and 2019
I	Single-incision scrotal orchiopexy	Surgical repositioning of the testis via scrotal approach
C	Traditional two-incision inguinal orchiopexy	Conventional inguinal surgical approach
O	Postoperative complications and surgical success	Short-term (wound dehiscence) and long-term (recurrence, atrophy, hydrocele, inguinal hernia) outcomes; identification of associated risk factors

**Table 2:** Characteristics of reviewed patients.

Patients—Total	362
Type of Diagnosis	
True Undescended Testis	236 (65.2%)
Retractile Testis—acquired UDT	126 (34.8%)
Operations	
Inguinal Orchidopexy	227 (48.3%)
Applied on true UDT	176 (37.4%)
Applied on RT—acquired UDT	51 (10.9%)
Scrotal Orchidopexy	216 (46%)
Applied on True UDT	48 (10.2%)
Applied on RT—acquired UDT	168 (35.7%)
Orchiectomy	2 (0.2%)
Laparoscopic Orchidopexy	24 (5.1%)
Inguinal Exploration	2 (0.4%)

**Table 3:** Results and complications of reviewed patients.

Type of Complication	Orchidopexies (n)
Atrophy	3
Recurrence	6
Hydrocele	2
Inguinal hernia	1
Wound dehiscence	4
Total	16
Type of cryptorchidism	
Undescended testis	13
Retractile testis	3
Type of orchidopexy	
Inguinal	10
Scrotal	6
Laparoscopic	0

**Table 4:** Inguinal/Scrotal orchidopexy and initial diagnosis.

Orchidopexy/ Diagnosis	True UDT	Retractile Testis	Total
Inguinal	176	51	227 (51.2%)
Scrotal	48	168	216 (48.8%)
Total	224	219	443

## Data Availability

The raw data supporting the conclusions of this article will be made available by the authors on request.
